# An energy minimization method for the correction of cupping artifacts in cone‐beam CT

**DOI:** 10.1120/jacmp.v17i4.6023

**Published:** 2016-07-08

**Authors:** Shipeng Xie, Wenqin Zhuang, Haibo Li

**Affiliations:** ^1^ Nanjing University of Posts and Telecommunications, College of Telecommunications & Information Engineering Nanjing Jiangsu China

**Keywords:** scatter correction, cupping artifacts, cone‐beam CT

## Abstract

The purpose of this study was to reduce cupping artifacts and improve quantitative accuracy of the images in cone‐beam CT (CBCT). An energy minimization method (EMM) is proposed to reduce cupping artifacts in reconstructed image of the CBCT. The cupping artifacts are iteratively optimized by using efficient matrix computations, which are verified to be numerically stable by matrix analysis. Moreover, the energy in our formulation is convex in each of its variables, which brings the robustness of the proposed energy minimization algorithm. The cupping artifacts are estimated as a result of minimizing this energy. The results indicate that proposed algorithm is effective for reducing the cupping artifacts and preserving the quality of the reconstructed image. The proposed method focuses on the reconstructed image without requiring any additional physical equipment; it is easily implemented and provides cupping correction using a single scan acquisition. The experimental results demonstrate that this method can successfully reduce the magnitude of cupping artifacts. The correction algorithm reported here may improve the uniformity of the reconstructed images, thus assisting the development of perfect volume visualization and threshold‐based visualization techniques for reconstructed images.

PACS number(s): 87.57.cp

## I. INTRODUCTION

An X‐ray system with a large‐area detector, which is commonly used for cone‐beam computed tomography (CBCT), is more susceptible to cupping artifacts generated by scatter and beam hardening. X‐ray scatter may lead to cupping artifacts, which is one of the most challenging problems in CBCT. Many correction methods that have been developed to reduce the cupping artifacts focus on reducing the scattered photons on the projective image. One such hardware‐based method involves physical scatter rejection techniques in the data acquisition system, such as the use of an air gap,^(1)^ antiscatter grid,^(2)^ or bowtie filter.^(3)^ The second category of software‐based approaches are based on Monte Carlo simulation,^4^, ^5^ which use Monte Carlo (MC) simulations to model the scatter distribution by tracing the history of photons from the source to the detector. Some improved Monte Carlo simulation algorithms, such as the GPU‐based method,^6^, ^7^ the combination of several variance‐reduction techniques,^(8)^ and the model‐based volume restoration approach,^(9)^ have been proposed in recent years. However, even using the fast algorithm, the formidably heavy computation load associated with Monte Carlo scatter estimation hinders its real‐life applications. The third category of methods estimates the contribution of scatter in the measured projection data and then subtracts it from the measured projection data, such as the beam‐stop array method,^(10)^ the moving blocker method,^(11)^ primary modula‐tion,^12^, ^13^, ^14^ artifact‐suppressed dictionary learning method,^15^, ^16^ and others.^17^, ^18^ However, these types of correction methods add extra hardware to the CBCT system, which can add difficulty and complexity. Although the image correction approach cannot be used to recover the contrast sensitivity, it is effective in recovering the accuracy of the projection image data for X‐ray transmission measurement and in reducing the nonuniformity of the CBCT signals that may be depicted as the cupping artifacts. All of these methods act directly on the projective image, requiring correction of every projected image. However, scatter correction performed on the projection images requires extensive computation to estimate the two‐dimensional scatter signal profile in conjunction with the use of sampled physical scatter measurements that are integrated with the image acquisition process or performed with additional exposures. We had observed that cupping artifacts can be corrected by using reconstructed image. Similar observations had also been reported in the literature.^19^, ^20^

In this study, we investigated an energy minimization method for cupping artifacts correction in the reconstructed image of CBCT. As we know, the cupping artifacts in reconstructed images are slow nonlinear varying. The reconstructed images can be decomposed into two parts: primary images and cupping artifacts images. To correct the cupping artifacts, we adapted a bias correction method previously developed for MR images.^(21)^ The robustness of the proposed algorithms was demonstrated in phantoms. This correction algorithm reported here can improve the uniformity of the reconstructed images, thus assisting the development of perfect volume visualization and threshold‐based visualization techniques for the reconstructed images.

## II. MATERIALS AND METHODS

### A. Decomposition of reconstructed image

Reconstruction images based on the work by Feldkamp et al.^(22)^ are popular in CBCT. The reconstruction image set can be equivalently written as

(1) $$\begin{array}{l} f=\frac{1}{4\pi^{2}}\int_{0}^{2\pi }\frac{d_{so}^{2}}{{\left(d_{so}+r\cdot s\right)}^{2}}\int_{-\infty }^{\infty }\frac{d_{so}}{{\left(d_{so}^{2}+t^{2}+z^{2}\right)}^{1/2}}\\ \cdot I_{3D}(t,z(r),\phi )\cdot \int_{-\infty }^{\infty }\left|\omega \right|e^{j2\pi \omega (t(r)-t)}d\omega dtd\phi \end{array}$$

where *d_so_* is the distance of the source‐to‐rotation axis, the coordinate along the detector that specifies the point of detection is $\left(t,z),I_{3D}(t,z(r),\phi \right)$ is the sequence of the projected image, *r* is extend from the origin to the reconstruction point, *s* is a unit vector along the ray from the source to the axis of rotation (i.e., normal to the detector), $z(r)=\frac{d_{so}(r\cdot z)}{d_{so}+r\cdot z}$, and $\int_{-\infty }^{\infty }\left|\omega \right|e^{j2\pi \omega (t(r)-t)}d\omega$ is the convolution function.

To address cupping artifacts in the reconstructed image, we formulate our method based on an image model that describes the composition of projected images, which are affected by scatter and beam hardening. A projected image can be modeled as

(2) $$I_{3D}=P_{3D}+S_{3D}+n$$

where $P_{3D}$ is the image derived from primary photons, $S_{3D}$ is the component caused by scatter and beam hardening, and *n* is additive noise with zero‐mean. The $P_{3D}$ measures an intrinsic physical property of the objects being imaged, which is therefore assumed to be piecewise (approximately) constant. We can prove that the reconstructed images based on the Feldkamp study^(22)^ can be written as follows:

(3) $$f=f_{p}+f_{s}+f_{n}$$

where $f_{p},f_{s}$ and $f_{n}$ are produced by $P_{3D},S_{3D}$, and noise *n*.

As in Eq. (2), a reconstruction image can be described as the true image plus the cupping artifacts. In the following section, we will give a method to remove from the reconstructed images.

### B. Representations of intrinsic components

To effectively use the properties of the $f_{s}$ and $f_{p}$, the $f_{s}$ is represented by a linear combination of a given set of smooth basis function $g_{1},L\;g_{M}$, which ensures the smoothly varying property of the cupping artifacts. The estimation of the scatter field is performed by finding the optimal coefficients $w_{1},L\;w_{M}$ in the linear combination $F_{s}(x)=\sum_{k=1}^{M}w_{k}g_{k}$. Consequently, the $f_{s}(x)$ can be expressed in the following vector form: $f_{s}(x)=w^{T}G(x)$, where $G(x)={\left(g_{1}(x),L\;g_{M}(x)\right)}^{T}$. In this paper, we use 10 polynomials as the smooth scatter function.

We presume that there are N types of tissues in the image domain $\Omega_{i}$. The true image f_p_(x) is virtually a constant $c_{i}$ for x in the i‐th tissue. We denote that the i‐th tissue is located in the region $\Omega_{i}$. Each $\Omega_{i}$ can be represented by its membership function $u_{i}$. In ideal conditions, the membership function $u_{i}$ is a binary membership function, with u_i_(x) = 1 for x∈Ω_i_ and u_i_(x) = 0 for x∈Ω_i_. Given the membership functions $u_{i}$ and constant $c_{i}$, the image $f_{p}$ can be expressed by

(4) $$f_{p}=\sum_{i=1}^{N}c_{i}u_{i}(x)$$

### C. Energy formulation for component optimization

We propose an energy minimization formulation previously developed for MR images. In this model, we take into account the problem of finding $f_{s}$ and $f_{p}$ of an observed postreconstructed image f such that the following energy is minimized

(5) $$F(f_{s},f_{p})=\int_{\Omega }{\left|f(x)-f_{p}(x)-f_{s}(x)\right|}^{2}dx$$

Clearly, if there are no constraints on the variables $f_{s}$ and $f_{p}$, minimization of F is an ill‐posed problem. Actually, the energy f=X−A[Y−B] is minimized by any variables $f_{s}$ and $f_{p}=f-f_{s}$ which are described in the Materials & Methods section B above. With these representations of the true image and the cupping artifacts, the energy f=X−A[Y−B] can be expressed as follow:

(6) $$F(f_{s},f_{p})=F(u,c,w)=\int_{\Omega }{\left|f(x)-\sum_{i=1}^{N}c_{i}u_{i}(x)-w^{T}G(x)\right|}^{2}$$

### D. Optimization of the cupping artifacts

A desirable property of this energy F(u,c,w) is that each variable, u, c, or w is convex. This property insures that F(u,c,w) has a unique minimum point in each of its variables.

For fixed c and u, we can minimize F(u,c,w). This can be achieved by solving the equation $\frac{\partial F}{\partial w}=0$. It can be shown that

(7) $$\frac{\partial F}{\partial w}=-2\int_{\Omega }G(x)\left|f(x)-\sum_{i=1}^{N}\left(c_{i}u_{i}(x)\right)\right|dx+2w\int_{\Omega }G(x)G{\left(x\right)}^{T}dx=0$$

The equation can be expressed as follow:

(8) Aw=v

where $A=\int_{\Omega }G(x)G{\left(x\right)}^{T}dx,\;v=\int_{\Omega }G(x)\left|f(x)-\sum_{i=1}^{N}(c_{i}u_{i}(x))dx\right|$

It is easy to show that the matrix A is nonsingular.^(21)^ Consequently, the vector $\hat{w}$ can be expressed as:

(9) $$\hat{w}=A^{-1}v$$

The estimated bias field is computed by

(10) $${\hat{f}}_{s}={\hat{w}}^{T}G(x)$$

For fixed w and u, the F(u,c,w) can be minimized with the variable c=c=^^^⁡(c1,L cN)T.

(11) $${\hat{c}}_{i}=\frac{\int_{\Omega }(f(x)-f_{s}(x))u_{i}(x)dx}{\int_{\Omega }u_{i}^{2}(x)dx},\;i=1,L,N$$

It can be shown that u=^^^⁡(u1,L uN)T is given by

(12) $${\hat{u}}_{i}=\{\begin{array}{l} 1,i=i_{\min}(x)\\ 0,i\neq i_{\min}(x) \end{array}$$

where $i_{\min}(x)=\arg\;\min\{f(x)-c_{i}-w^{T}G(x)\}$.

### E. Implementation

**Figure 1:**
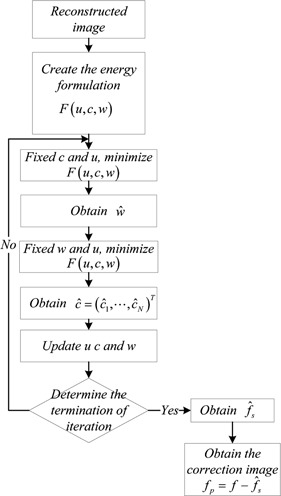
The process of the proposed cupping artifacts correction.

From the previous sections, we summarize the scheme of minimization of the energy F(u,c,w) as shown in Fig. 1.

$\left|c^{\left(n\right)}-c^{\left(n-1\right)}\right|<\varepsilon$ is the convergence criterion, where $c^{\left(n\right)}$ is updated from the vector c at the n‐th iteration, and ε is assigned to 0.001. The F(u,c,w) rapidly declines and meets the minimum value in less than 15 iterations in our applications.

### F. Evaluation of cupping correction

Quantitative image quality parameters were analyzed according to the work by Jo et al.^(14)^ The magnitude of cupping $\tau_{\text{cup}}=100(u_{M,\text{edge}}-u_{M,\text{center}})/u_{M,\text{edge}}$ was extracted in terms of voxel values at the center $u_{M,\text{center}}$ and edge $u_{M,\text{edge}}$ of the water phantom.

Root mean square contrast (RMSC) is defined as the standard deviation of the pixel intensities:

(13) $$RMSC=\sqrt{\frac{1}{MN}\sum_{i=o}^{N-1}\sum_{j=0}^{M-1}{\left(I_{ij}-\bar{I}\right)}^{2}}$$

where $I_{ij}$ is the *i‐th j‐th* element of the two‐dimensional image of size *M* by *N* and $\bar{I}$ is the average intensity of all pixel values in the image. The image *I* is assumed to have its pixel intensity normalized in the range [0,255].

## III. RESULTS

### A. Skull phantom

**Figure 2:**
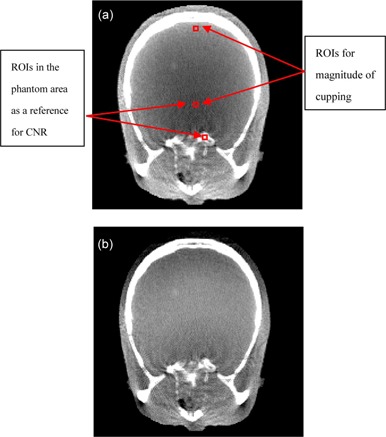
Sample axial views selected from reconstructed images of the skull phantom before (a) and after cupping artifact correction (b).

**Figure 3:**
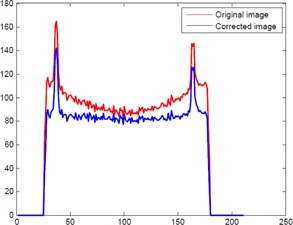
The 1D horizontal profile of the measured skull phantom: The column is the profiles of the images, which are at middle row in Fig. 2.

The experimental data were obtained from Rezvani et al.^(23)^ The image size is 211×211. After correction, the images presented significantly improved signal uniformity, as shown in Fig. 2 and Fig. 3. For a quantitative analysis of the reconstructed images, we measure the magnitude of cupping in the selected ROIs, as shown in Fig. 2(a). The analyses are given in Table 1. The correction process take 1.89 s for a slice (CPU: i5–2450, RAM: 6GB, GPU: NVIDA GeForce 610M).

The 1D horizontal profile before and after correction can be seen in Fig. 3. As before, a great reduction in the cupping artifact is observed in the corrected image.

**Table 1:** Quantitative analysis of a skull phantom. Reconstructed image before correction of cupping artifacts (RI_BC), reconstructed image after correction of cupping artifacts (RI_AC)

	*RMSE (edge)*	*RMSE (center)*	$\tau_{cup}$ (%)	*CNR*
RI_BC	2.22	1.89	16.44	5.61
RI_AC	2.26	1.68	8.9	9.52

### B. CTP486 phantoms

**Figure 4:**
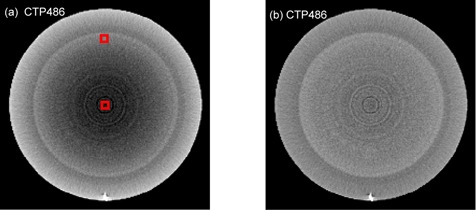
Sample axial views selected from two different slices of reconstructive images of the CTP486 in the CatPhan500 are shown before (a) and after scatter correction (b). The images are rescaled to the same size such that they can be displayed in the same figure and are displayed at the same gray scale. ROIs for magnitude of cupping are marked with red blocks.

CTP486 (The Phantom Laboratory, Salem, NY) is used in this experiment. Figure 4 shows the reconstructed images. The CTP486 module is cast from a uniform material that has a CT number within 2% (0–20H) of water. The image size is 229×229. The correction process take 1.93 s for a slice (CPU: i5–2450, RAM: 6GB, GPU: NVIDA GeForce 610M).

ROIs for magnitude of cupping are shown in Fig. 4(a). Our approach clearly reduces the $\tau_{cup}$ magnitude by an average of 95%.

**Figure 5:**
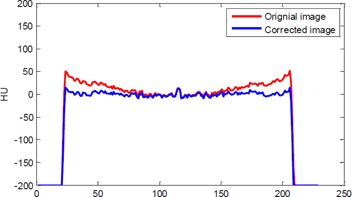
The 1D horizontal profile of the measured phantom. The column is the profiles of the images, which are at middle row in Fig. 4.

The 1D horizontal profile before and after correction can be observed in Fig. 5.

### C. A mouse bone

**Figure 6:**
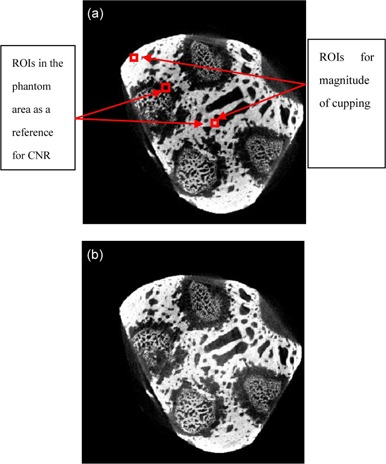
Sample axial views selected from the reconstructive images of the mouse's bone are shown before (a) and after scatter correction (b).

A mouse's bone scan data are obtained from a Micro‐CT called Hiscan‐M1000 (Hays Feder Information Technology Co., Suzhou, China). The acquisitions for the reconstructed images consist of 360 projections, with 80 kVp, 200 uA, 30 ms. Sample axial views selected from the reconstructive images of the mouse bone is shown before (Fig. 6(a)) and after scatter correction (Fig. 6(b)). The image size is 339×339. The correction process takes 2.7 s for a slice (CPU: i5–2450, RAM: 6GB, GPU: NVIDA GeForce 610M). We measure the magnitude of cupping in the selected ROIs, as shown in Fig. 6(a). Our approach reduces the $\tau_{cup}$ from 23.8% to 9.8%. The quantitative image quality analyses are provided in Table 2.

**Table 2:** Quantitative analysis of a mouse bone. Reconstructed image before correction of cupping artifacts (RI_BC), reconstructed image after correction of cupping artifacts (RI_AC)

	*RMSE (edge)*	*RMSE (center)*	$\tau_{cup}$ (%)	*CNR*
RI_BC	6.36	3.57	28.8	11.97
RI_AC	6.49	3.57	9.8	15.87

### D. Pelvis patient

**Figure 7:**
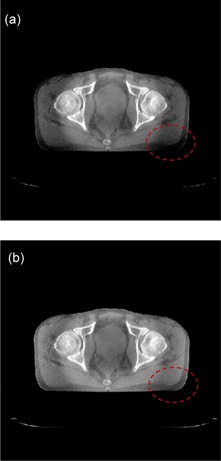
Sample axial views selected from two different slices of the pelvis CBCT image are shown before (a) and after scatter correction (b). The images are displayed in the same window.

The proposed correction method is further evaluated on the CBCT images acquired from a pelvis patient. The tabletop system operates in the full‐fan scan mode and a data acquisition in a 360° scan. After correction, the images presented significantly improved signal uniformity, as shown in Fig. 7.

### E. Comparison with another method

**Figure 8:**
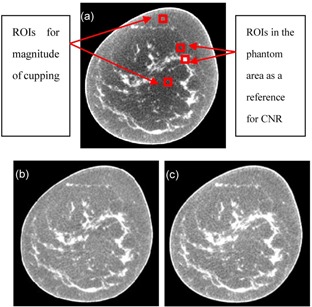
Comparison of breast CT images before (bottom left) and after (top) cupping artifact correction, using the method developed by Yang et al.^(19)^ (Bottom right) This paper's method.

In this study, our idea is originated from MRI. We find another cupping artifact method inspired by the MR bias correction method from the work by Yang et al.^(19)^ The results are shown in Fig. 8. For a quantitative analysis of the reconstructed images, we measure the magnitude of cupping and CNR in the selected ROIs, and the quantitative analyses are given in Table 3.

**Table 3:** Reconstructed image before correction of cupping artifacts (RI_BC), reconstructed image after correction of cupping artifacts, from work by Yang et al.^(19)^ RI_AC_Ref19, reconstructed image after correction of cupping artifacts using this method (RI_AC_our)

	*RMSE (edge)*	*RMSE (center)*	$\tau_{cup}$ (%)	*CNR*
RI_BC	4.42	5.37	22.27	11.48
RI_AC_Ref19	5.31	4.41	0.6	7.74
RI_AC_our	4.40	5.8	0.7	8.59

## IV. DISCUSSION & CONCLUSIONS

For a quantitative analysis of the reconstructed images, we measure the magnitude of cupping in the selected ROIs for skull phantom, shown in Fig. 2. The analyses are given in Table 1. Our approach clearly reduces the $\tau_{cup}$ magnitude by an average of 50%. It can be seen that the proposed correction method works very well. As shown in Figs. 3 and 5 and Tables 1 and 2, the proposed method markedly reduced the magnitude of cupping, increased the CNR of CBCT, and demonstrated no obvious change of the RMSEs. In other words, the image quality was improved after cupping artifact correction.

Our approach clearly reduces the $\tau_{cup}$ magnitude by an average of 95% in the CTP486 phantom. As shown in Fig. 4, a marked reduction in cupping artifacts is observed in the corrected image.

As shown in Fig. 7, the proposed method can significantly improved signal uniformity and the image quality. The results clearly show that the proposed correction method works very well.

In Yang et al.,^(19)^ the method automatically corrects the cupping artifacts using a nonpara‐metric coarse‐to‐fine approach which allows the cupping artifacts to be modeled with different frequency ranges and without user supervision. Figure 8 shows that our method equally for the correction of cupping artifacts, as well as that detailed in Yang et al. Our method has a higher average CNR of 8.59 (Fig. 8(c)), which is higher than the previously reported average CNR (Fig. 8(b)) by 1.11‐fold.

In Reitz et al.,^(24)^ a scatter correction method is based on a superposition of precalculated Monte Carlo generated pencil beam scatter kernels. The cupping artifacts of water phantom are reduced from 20% without scatter correction to 4% with scatter correction (reduces the $\tau_{cup}$ magnitude 80%). In our method, the cupping artifacts of CTP486 (water phantom) is reduced by about 95%.

As our correction method involves processing of the reconstructed images only, it does not require any hardware modifications for signal measurements. The correction algorithm reported here may improve the uniformity of the reconstructed images, thus assisting in the development of perfect volume visualization and image segmentation techniques.

## ACKNOWLEDGMENTS

This work was supported by the National Natural Science Foundation of China (Grant No. 11547155 and No. 81530060), National Natural Science Foundation of Jiangsu Province (Grant No. BK20130883), the NUPTSF (Grant No. NY213011 and No. NY214026) and the National High‐tech R&D Program for Young Scientists by the Ministry of Science and **Technology of China** (863 **Program, Grant No. 2015AA020917).**

## COPYRIGHT

This work is licensed under a Creative Commons Attribution 3.0 Unported License.
